# The prognostic value of the albumin/neutrophil-to-lymphocyte ratio in colorectal cancer patients: a retrospective cohort study

**DOI:** 10.3389/fonc.2026.1668103

**Published:** 2026-03-26

**Authors:** Hailun Xie, Nuo Xu, Lishuang Wei, Shuangyi Tang, Jialiang Gan

**Affiliations:** 1 Department of Gastrointestinal and Gland Surgery, The First Affiliated Hospital, Guangxi Medical University, Nanning, Guangxi, China; 2 Guangxi Key Laboratory of Enhanced Recovery After Surgery for Gastrointestinal Cancer, The First Affiliated Hospital, Guangxi Medical University, Nanning, Guangxi, China; 3 Department of Pharmacy, The First Affiliated Hospital, Guangxi Medical University, Nanning, Guangxi, China; 4 Department of Colorectal and Anal Surgery, The First Affiliated Hospital, Guangxi Medical University, Nanning, Guangxi, China

**Keywords:** Albumin/neutrophil-to-lymphocyte ratio, colorectal cancer, complications, overall survival, progression-free survival, sarcopenia

## Abstract

**Background:**

The Albumin/Neutrophil-to-Lymphocyte Ratio (ANLR) integrates inflammatory and nutritional pathways, yet its prognostic utility in colorectal cancer (CRC) remains underexplored. This study investigates the association between ANLR and progression-free survival (PFS) as well as overall survival (OS) in CRC patients, aiming to clarify its clinical significance and utility in treatment decision-making.

**Methods:**

This retrospective cohort study included 1,436 CRC patients who underwent surgical resection at a single institution between 2015 and 2017. Survival curves for PFS and OS were generated using the Kaplan-Meier method, with differences compared via log-rank tests. Cox proportional hazards regression models were used to evaluate the relationship between ANLR and survival outcomes, while logistic regression analysis assessed the independent association of ANLR with sarcopenia and postoperative complications. Nomograms incorporating ANLR and other significant prognostic factors were constructed to predict 1-, 3-, and 5-year survival rates. The clinical utility of these models was validated using decision curve analysis (DCA) against traditional TNM staging.

**Results:**

The median follow-up duration was 65 months (interquartile range: 41–78 months). Patients with low ANLR (<15.51) had significantly poorer 5-year PFS (48.2% vs. 63.0%, p < 0.001) and OS (50.9% vs. 65.8%, p < 0.001) compared to those with high ANLR (≥15.51). ANLR demonstrated superior predictive efficacy for outcomes compared to other inflammation-nutrition indices. Multivariate Cox regression identified high ANLR as an independent predictor of improved PFS (hazard ratio [HR] = 0.745, 95% CI: 0.630–0.880, p = 0.001) and OS (HR = 0.739, 95% CI: 0.622–0.878, p = 0.001). Additionally, high ANLR was independently associated with a 40.7% lower risk of sarcopenia (Odds Ratio [OR] = 0.593, 95% CI: 0.442–0.796, p < 0.001) and a reduced risk of complications (OR = 0.564, 95% CI: 0.429–0.742, p < 0.001). The ANLR-based nomograms showed high predictive accuracy (C-indices: 0.719 for PFS, 0.727 for OS) and outperformed TNM staging, confirming greater clinical utility.

**Conclusion:**

ANLR is a promising prognostic biomarker for predicting PFS and OS in CRC patients, with additional value in assessing sarcopenia and complication risks. ANLR-based nomograms provide a valuable tool for personalized survival prediction, supporting tailored treatment strategies to improve patient outcomes.

## 1 Introduction

Colorectal cancer (CRC) is a prevalent malignancy posing a significant threat to global health, characterized by high incidence and mortality rates. According to the latest global cancer statistics, CRC ranks third in newly diagnosed cases and second in cancer-related deaths (1). Such high burden underscores its profound impact on the quality of life and life expectancy of affected individuals. Patients with CRC often face multiple challenges, including the physical and emotional toll of the disease, adverse treatment effects, and recurrence risk. The consequences extend beyond individuals to affect families and healthcare systems alike, highlighting the urgent need for effective strategies in prevention, early detection, and treatment to improve outcomes and alleviate CRC’s public health burden (2). While advances in CRC diagnosis and treatment—such as refined surgical techniques, novel chemotherapeutic agents, and the emergence of targeted and immunotherapies—are promising, many patients are still diagnosed at intermediate or advanced stages. This late diagnosis frequently limits opportunities for optimal treatment, leading to poor prognoses. Strengthened screening programs and public awareness initiatives are therefore critical to promoting early detection and enhancing patient outcomes (3). In China, the 5-year survival rate for CRC patients is only 56.9%, relatively low compared to neighboring Asia-Pacific regions. This figure underscores critical challenges in CRC management, including late-stage diagnosis and limited access to advanced treatments. Improving survival will require enhanced early detection, better access to effective therapies, and public education campaigns focused on health promotion and screening (4, 5). Identifying reliable prognostic biomarkers is thus crucial for predicting disease progression, evaluating treatment efficacy, and developing personalized treatment plans.

Traditional prognostic indicators—including TNM staging, tumor type, and degree of differentiation—are widely used in clinical practice but have limitations in accurately predicting individual outcomes. These conventional assessments often fail to fully capture tumors’ biological complexity or variability in treatment responses (6), emphasizing the need for advanced biomarkers to improve prognostic accuracy and enable personalized strategies. Recent studies increasingly recognize systemic inflammation and nutritional status as key factors influencing cancer progression and outcomes, with impacts on tumor growth, metastasis, and treatment response. These insights underscore the value of biomarkers that reflect the systemic environment to inform clinical decision-making (7–9).

The albumin/neutrophil-to-lymphocyte ratio (ANLR), which integrates the neutrophil-to-lymphocyte ratio (NLR) and albumin, represents a novel composite index for evaluating inflammation and nutrition in CRC. Previous research has demonstrated a strong correlation between NLR and systemic inflammation, supporting its use as a predictive marker of inflammatory status. An elevated NLR indicates heightened inflammation, associated with poorer clinical outcomes, and it is widely utilized across oncology, diabetes, and cardiovascular diseases as a valuable biomarker for assessing severity and predicting outcomes (10–12). NLR also serves as an independent risk factor for CRC onset and progression while effectively reflecting patients’ inflammatory levels (13, 14). Albumin, a key clinical indicator of nutritional status, is strongly linked to recurrence and poor survival in CRC patients. In the context of systemic inflammation, hepatic protein synthesis undergoes reprogramming, with the liver prioritizing acute-phase protein production—thus, albumin has recently been proposed as a marker to characterize systemic inflammation in cancer patients (15). Low albumin levels typically reflect malnutrition and systemic inflammation, both of which impair treatment response and recovery, and albumin has been reported as an effective indicator for assessing disease progression and prognosis in cancer patients (16, 17).

By integrating inflammatory and nutritional biomarkers, ANLR provides a comprehensive assessment of cancer patients’ systemic status, illuminating the interplay between these pathways and clinical outcomes—thus establishing its utility for prognostic evaluation and therapeutic guidance. Previous studies have validated ANLR’s prognostic capacity across diverse conditions: elevated peripheral ANLR effectively predicts adverse outcomes in coronary artery disease and diabetic foot ulcers (18–20). This predictive power extends to gastrointestinal cancers, as demonstrated by Onuma et al., who identified preoperative ANLR as a significant prognostic indicator for gastric cancer patients following curative gastrectomy (21). Nevertheless, the precise mechanisms and clinical applicability of ANLR in colorectal cancer remain incompletely characterized, warranting further investigation.

Therefore, this study aims to retrospectively analyze clinical data from CRC patients to examine the relationship between ANLR and progression-free survival (PFS) and overall survival (OS), offering new insights to improve prognostic assessment and clinical management in CRC.

## 2 Materials and methods

### 2.1 Study population

This retrospective analysis enrolled 1,436 CRC patients who underwent surgical resection at the First Affiliated Hospital of Guangxi Medical University between 2015 and 2017. The inclusion criteria were as follows: histological confirmation of CRC; availability of complete data on albumin, neutrophil percentage, and other relevant clinicopathological factors; and age ≥ 18 years.​ Exclusion criteria included: prior neoadjuvant chemotherapy before surgery; concurrent malignancies; pre-existing autoimmune diseases; acute or chronic inflammatory conditions that might affect neutrophil or albumin levels at the time of data collection; lack of follow-up data; and incomplete medical records.​ This study was approved by the Institutional Review Board of the First Affiliated Hospital of Guangxi Medical University, and informed consent was obtained from all participants.

### 2.2 Data collection

Clinicopathological data were comprehensively collected from the hospital’s electronic medical record system. Specifically, this included patients’ baseline information: gender, age, height, weight, body mass index (BMI), presence of hypertension, presence of diabetes, and contact details. Tumor-related information encompassed tumor location (colon or rectum), TNM staging (determined in accordance with the 8th Edition of the American Joint Committee on Cancer (AJCC) staging system), tumor size, differentiation grade, perineural invasion, and vascular invasion. All laboratory parameters were measured under fasting conditions within 1 week before surgery, including neutrophil count, platelet count, lymphocyte count, albumin levels, and carcinoembryonic antigen (CEA) levels. The neutrophil-to-lymphocyte ratio (NLR) was calculated as neutrophil count (×10^9^/L) divided by lymphocyte count (×10^9^/L). The platelet-to-lymphocyte ratio (PLR) was defined as platelet count (×10^9^/L) divided by lymphocyte count (×10^9^/L). The Prognostic Nutritional Index (PNI) was defined as albumin (g/L) +5×lymphocytes (×10^9^/L). The ANLR was computed using the formula: ANLR = Albumin (g/L)/NLR.

### 2.3 Patients’ follow-up and outcomes

Follow-up assessments were scheduled every 3 months for the first 2 years, every 6 months for the subsequent 3 years, and annually thereafter. Each follow-up included a detailed inquiry into symptoms and signs to detect potential recurrence or metastasis, supplemented by essential examinations: routine blood tests, biochemical analyses, tumor marker measurements, colonoscopy, and imaging studies (CT or MRI) for a comprehensive health evaluation.​ All 1,436 eligible patients from the First Affiliated Hospital of Guangxi Medical University (treated between 2015 and 2017) underwent long-term follow-up via telephone interviews and outpatient visits. The follow-up duration ranged from 1 to 107 months (mean: 61 months; median: 65 months; interquartile range: 41–78 months). Recurrence was confirmed by imaging and pathological findings. During the follow-up period, 580 patients (40.4%) died, and 398 (27.2%) experienced recurrence.​ The primary endpoints were progression-free survival (PFS) and overall survival (OS). PFS was defined as the time from surgical resection to the first occurrence of local recurrence, distant metastasis, or death. OS was measured as the time from surgery to death from any cause or the date of the last follow-up. Sarcopenia was diagnosed in accordance with the criteria established by the Asian Working Group for Sarcopenia (AWGS 2019): skeletal muscle mass index (SMI) < 6.92 kg/m² for males and < 5.13 kg/m² for females. According to previous literature (22), the SMI derived from the anthropometric equation was calculated as follows: [0.193 × weight (kg) + 0.107 × height (cm) - 4.157 × sex (1 = male, 2 = female) - 0.037 × age (year) - 2.631]/height squared (m²). For postoperative complications (modified Clavien Classification System), the time window was defined as 90 days after surgery, and we analyzed complications of Grade II or higher.

### 2.4 Statistical analysis

Statistical analyses were performed using SPSS 25.0 and R 4.0.2 software. Continuous variables were presented as mean ± standard deviation (SD) or median (interquartile range, IQR), while categorical variables were expressed as counts and percentages. The optimal cutoff value for ANLR was determined via receiver operating characteristic (ROC) curve analysis. Kaplan–Meier survival curves combined with log-rank tests were used to compare survival outcomes between the low- and high-ANLR groups. Cox proportional hazards models with three-knot restricted cubic spline (RCS) were used to illustrate ANLR as a continuous predictor of survival. Cox proportional hazards models were employed to identify independent prognostic factors. Logistic regression analysis was employed to determine the independent association between ANLR and sarcopenia/complications in CRC patients. Nomograms were constructed based on the significant variables identified in the Cox regression model using the rms package in R. The predictive performance of the nomograms was evaluated using the concordance index (C-index) and calibration curves. Decision curve analysis (DCA) was used to compare the clinical utility of the ANLR-based nomogram with that of the traditional TNM staging system. A two-tailed p-value < 0.05 was considered statistically significant.

## 3 Results

### 3.1 Demographic characteristics

This retrospective study analyzed 1,436 CRC patients with a median age of 58.17 years (SD: 13.12), including 903 males (62.9%). The cohort exhibited a median BMI of 22.04 kg/m² (IQR: 19.96–24.35), with preoperative comorbidities comprising hypertension (16.7%, n=240) and diabetes (6.1%, n=88). Tumor profiling revealed advanced disease: 74.4% (n=1,069) presented T3–T4 stage lesions, 47.0% (n=675) had TNM stage III–IV disease, and 9.5% (n=136) showed distant metastases (M1). Nodal involvement distribution was N0 (56.0%, n=804), N1 (27.6%, n=397), and N2 (16.4%, n=235). Perineural and vascular invasion occurred in 10.4% (n=149) and 17.2% (n=247) of cases, respectively, while 51.0% (n=733) had rectal tumors. Pathologically, 13.1% (n=188) demonstrated poor differentiation. Systemic biomarker assessment showed median CEA levels of 3.87 ng/mL (IQR: 2.07–10.73). Treatment modalities included radiotherapy (9.3%, n=133) and chemotherapy (45.6%, n=655). During follow-up, mortality and recurrence rates reached 40.4% (n=580) and 27.2% (n=398), respectively. Resource utilization metrics indicated a median hospitalization duration of 17.00 days (IQR: 11.00–21.00) with associated costs of ¥49,541.85 (IQR: ¥44,564.47–55,983.79).

### 3.2 Determination of ANLR cutoff value and group comparisons

The optimal ANLR cutoff was established at 15.51 (AUC = 0.582; sensitivity = 0.644, specificity = 0.507), which we used to stratify 1,436 patients into low ANLR (<15.51, n=599) and high ANLR (≥15.51, n=837) groups (**Supplementary Figure 1**). The bootstrap-corrected C-index was 0.561, and the bootstrap-corrected calibration slope was 0.679. The distribution of 1,000 Bootstrap-derived cut-off values was concentrated around the original threshold (**Supplementary Figure 2**), with no significant skewness, indicating minimal single-center bias. Notably, lower ANLR values demonstrated significant associations with multiple adverse clinical features: male gender, advanced age, reduced BMI, hypertension, poorly differentiated tumors, rectal tumor location, larger tumor diameter, elevated CEA levels, prolonged hospitalization, and higher treatment costs. Critically, the low ANLR group exhibited substantially worse clinical outcomes, with significantly higher mortality (49.1% vs. 34.2%, p<0.001) and recurrence rates (31.4% vs. 25.1%, p<0.001) compared to the high ANLR group (**Table 1**).

**Table 1:** Association between ANLR and PFS of CRC patients.

ANLR	Model a	p value	Model b	p value	Model c	p value
Continuous (per SD)	0.836 (0.77,0.908)	<0.001	0.912 (0.84,0.991)	0.029	0.905 (0.831,0.986)	0.023
Cutoff value (High)	0.653 (0.558,0.765)	<0.001	0.762 (0.648,0.896)	0.001	0.745 (0.630,0.880)	0.001
Quartiles						
Q1 (~11.70)	ref		ref		ref	
Q2 (11.70~17.31)	0.817 (0.663,1.007)	0.058	0.872 (0.707,1.075)	0.2	0.832 (0.671,1.032)	0.094
Q3 (17.31~24.98)	0.634 (0.508,0.791)	<0.001	0.714 (0.57,0.893)	0.003	0.699 (0.555,0.879)	0.002
Q4 (24.98~)	0.635 (0.509,0.793)	<0.001	0.788 (0.628,0.99)	0.041	0.771 (0.609,0.977)	0.031
p for trend		<0.001		0.009		0.009

Model a: No adjusted.

Model b: Adjusted for gender, age, and BMI.

Model c: Adjusted for gender, age, BMI, hypertension, diabetes, T stage, N stage, M stage, tumor size, perineural invasion, vascular invasion, differentiation, radiotherapy, chemotherapy.

### 3.3 Comparative prognostic performance of ANLR versus established inflammation-nutrition indices

Spearman correlation analysis showed moderate correlations between ANLR and established inflammation-nutrition indices (NLR, PLR, PNI), especially with PNI (**Supplementary Figure 3**), which may be attributed to overlapping components of the indicators. We conducted ROC analysis to evaluate the prognostic utility of the ANLR against established biomarkers—NLR, PLR, and PNI. Consistently across survival endpoints, ANLR demonstrated superior discriminative ability. For PFS, ANLR achieved higher AUC values than comparator indices at both 3 years (0.553 vs. NLR: 0.544, PLR: 0.533, PNI: 0.556) (**Supplementary Figure 4A**) and 5 years (0.554 vs. NLR: 0.545, PLR: 0.540, PNI: 0.559) (**Supplementary Figure 4B**). This performance advantage extended to OS prediction, where ANLR similarly outperformed other markers at 3-year follow-up (AUC: 0.571 vs. NLR: 0.565, PLR: 0.555, PNI: 0.566) (**Supplementary Figure 4C**) and maintained superior accuracy at 5 years (0.559 vs. NLR: 0.551, PLR: 0.544, PNI: 0.561) (**Supplementary Figure 4D**). C-index analysis supplemented with the “ANLR + traditional inflammation-nutrition indices” combined model revealed that the AUC of the combined model was slightly higher than that of single traditional indices. Although the difference was not statistically significant, it confirmed that ANLR can provide incremental predictive information rather than merely duplicating the value of existing indicators (**Supplementary Tables 2**, **S3**).

### 3.4 Survival differences between low-ANLR and high-ANLR groups

**Figure 1:**
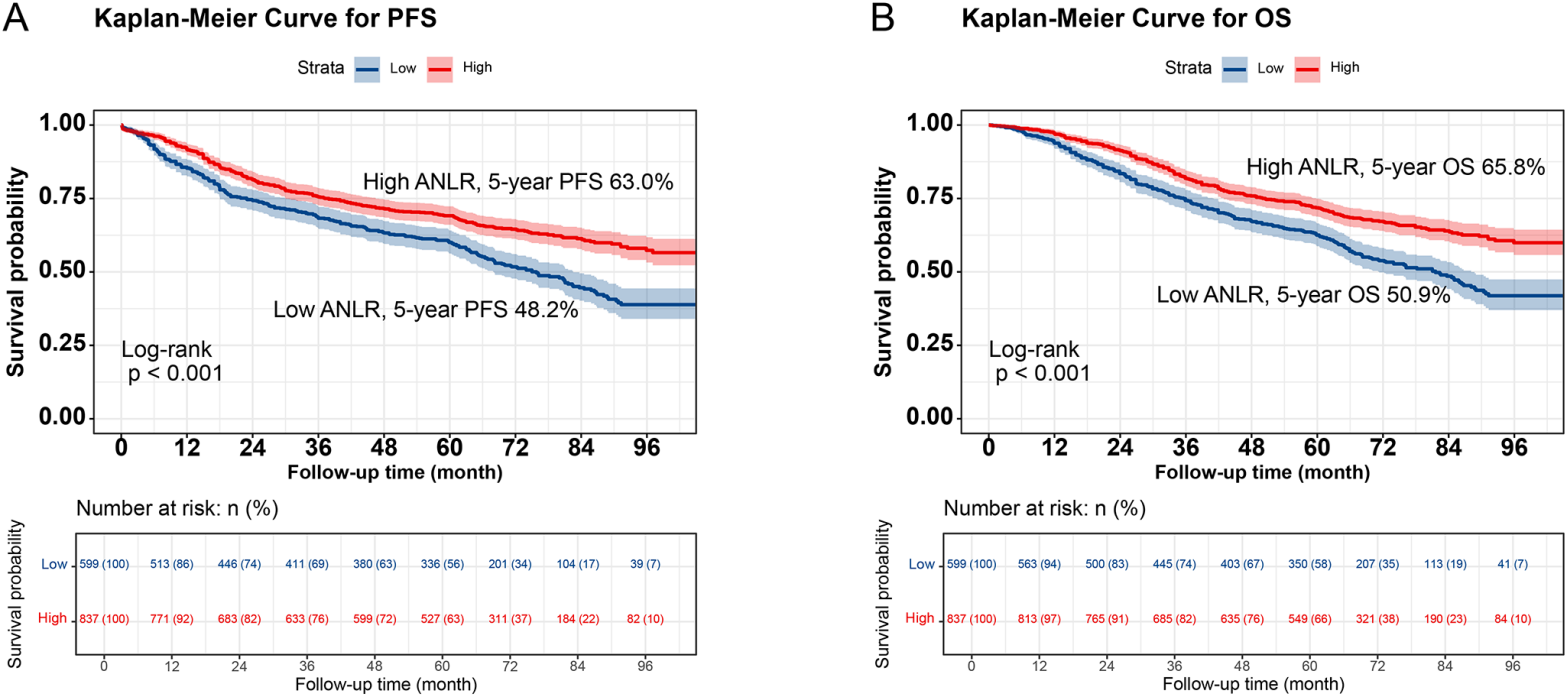
Kaplan-Meier curve of ANLR in CRC patients. **(A)**, Progression-free survival; **(B)**, Overall survival.

**Figure 2:**
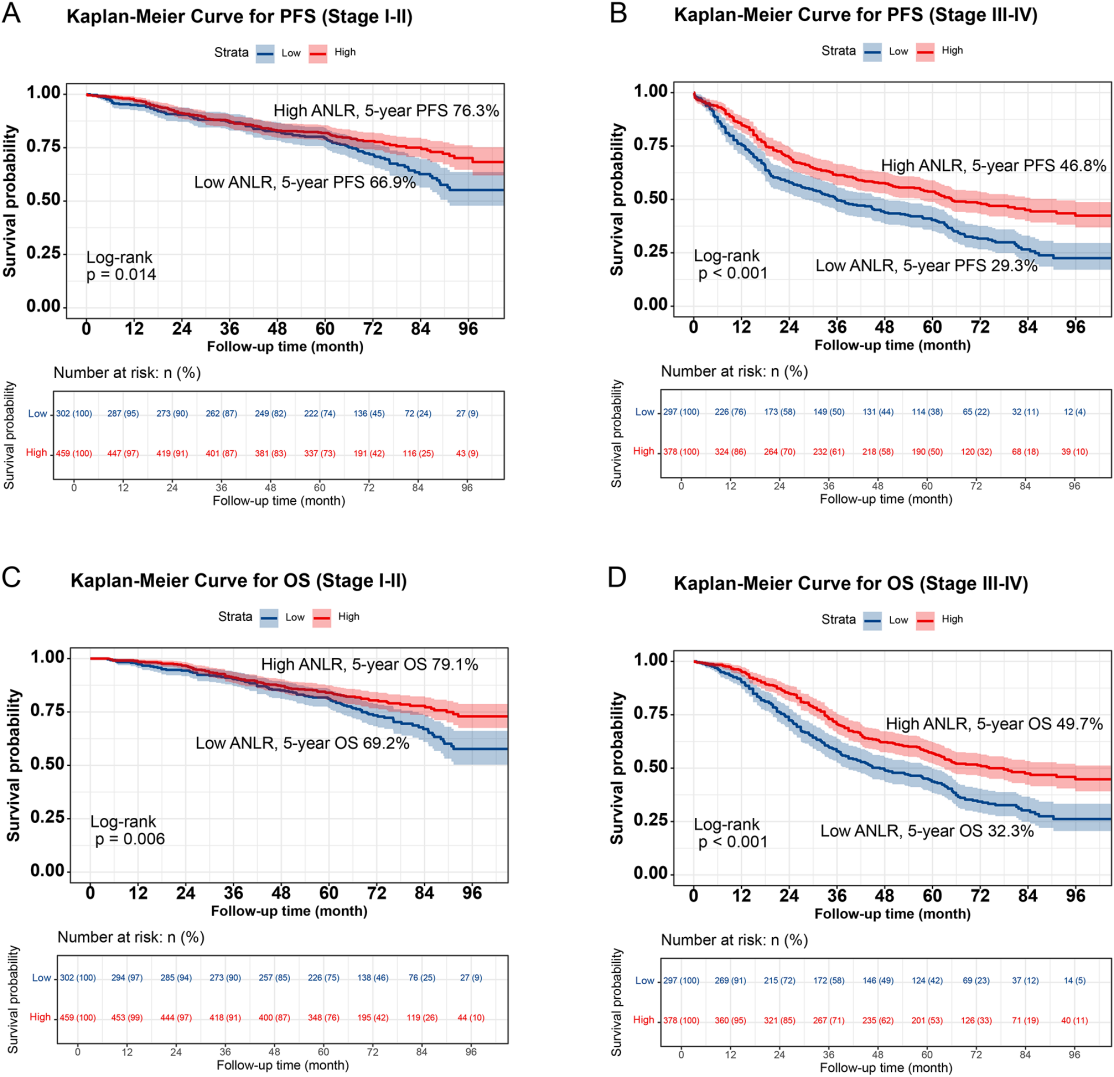
Stratified Kaplan-Meier curve of ANLR based on TNM stage subgroup in CRC patients. **(A)**, Progression-free survival (Stage I-II); **(B)**, Progression-free survival (Stage III-IV); **(C)**, Overall survival (Stage I-II); **(D)**, Overall survival (Stage III-IV).

Kaplan-Meier analysis demonstrated significantly worse survival outcomes in low-ANLR patients versus high-ANLR counterparts, with markedly reduced 5-year rates for both PFS (48.2% vs. 63.0%, p<0.001) and OS (50.9% vs. 65.8%, p<0.001; **Figures 1A, B**). Critically, this prognostic pattern persisted across all subgroups. In TNM staging subgroups, low-ANLR patients exhibited compromised survival regardless of disease stage (Stage I-II: PFS 66.9% vs. 76.3%, p=0.014; OS 69.2% vs. 79.1%, p=0.006; Stage III-IV: PFS 29.3% vs. 46.8%, OS 32.3% vs. 49.7%, both p<0.001; **Figures 2A–D**). Similarly, for tumor location subgroups, colon cancer patients with low ANLR had inferior PFS (43.9% vs. 61.5%, p<0.001) and OS (46.6% vs. 65.4%, p=0.001) (**Supplementary Figures 5A, B**), while rectal cancer patients showed parallel deficits (PFS: 51.4% vs. 65.0%, OS: 54.0% vs. 66.4%, both p<0.001) (**Supplementary Figures 5C, D**).

### 3.5 Relationships between ANLR and survival outcomes

**Figure 3:**
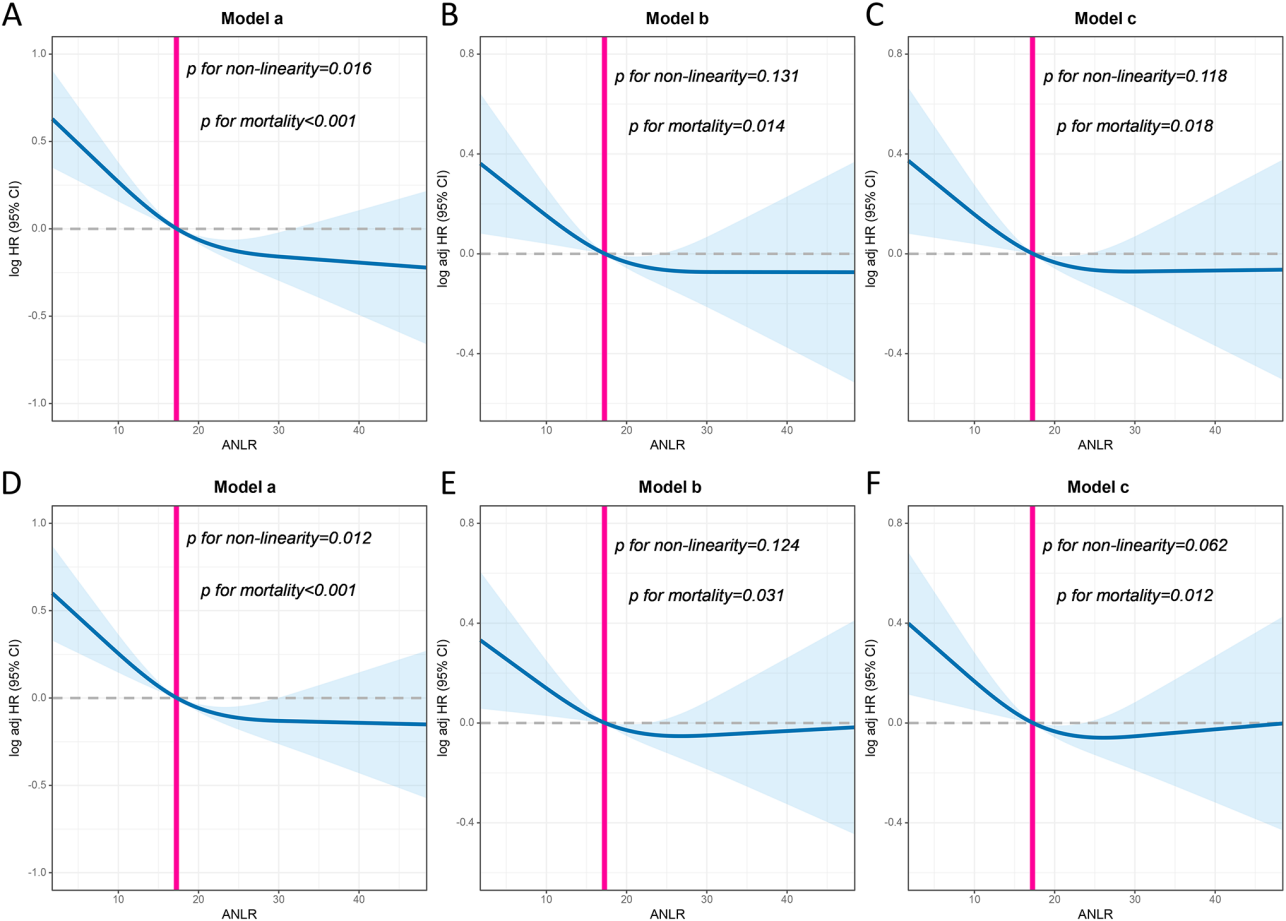
The association between ANLR and survival in CRC patients. **(A–C)**, Progression-free survival; **(D–F)**, Overall survival. Model a: Unadjusted. Model b: Adjusted for gender, age, and BMI. Model c: Adjusted for gender, age, BMI, hypertension, diabetes, T stage, N stage, M stage, tumor size, perineural invasion, vascular invasion, differentiation, radiotherapy, chemotherapy.

A non-linear relationship was found between ANLR and PFS/OS risk, revealing an L-shaped pattern (p < 0.001). As the ANLR increased, the HR gradually decreased (**Figure 3**). Multivariate Cox regression analysis revealed that for every 1 standard deviation (SD) increase in ANLR, the risk of adverse PFS in CRC patients decreased by 9.5% (HR = 0.905, 95% CI: 0.831–0.986, p = 0.023). Compared with patients in the low-ANLR group, those in the high-ANLR group had a significantly lower risk of adverse PFS (HR = 0.745, 95% CI: 0.630–0.880, p = 0.001). Quartile analysis of ANLR showed that patients in the second, third, and fourth quartiles had adverse PFS rates 0.817, 0.634, and 0.635 times those of patients in the first quartile, respectively (**Table 1**).​ For OS, multivariate Cox regression analysis indicated that for every 1 SD increase in ANLR, the risk of adverse OS decreased by 9.5% (HR = 0.905, 95% CI: 0.828–0.988, p = 0.026). The low-ANLR group had a 26.1% higher risk of adverse OS than the high-ANLR group (HR = 0.739, 95% CI: 0.622–0.878, p = 0.001). As ANLR increased, the HR for adverse OS gradually decreased: quartile 2 (Q2: 0.787), quartile 3 (Q3: 0.632), and quartile 4 (Q4: 0.602) were all associated with a reduced risk of adverse OS (**Table 2**). Sensitivity analysis showed that excluding patients who died within 3 months after surgery did not alter the prognostic effect of ANLR (**Supplementary Tables 4**, **S5**). Multivariable forest plot analysis demonstrated that ANLR served as an independent prognostic factor for PFS across most patient subgroups (**Supplementary Figure 6A**). Similarly, in terms of OS, patients with low ANLR generally had poorer prognoses compared to those with high ANLR in the majority of subgroups (**Supplementary Figure 6B**).

**Table 2:** Association between ANLR and OS of CRC patients.

ANLR	Model a	p value	Model b	p value	Model c	p value
Continuous (per SD)	0.822 (0.755,0.895)	<0.001	0.901 (0.827,0.981)	0.016	0.905 (0.828,0.988)	0.026
Cutoff value (High)	0.634 (0.539,0.746)	<0.001	0.738 (0.625,0.871)	<0.001	0.739 (0.622,0.878)	0.001
Quartiles						
Q1 (~11.70)	ref		ref		ref	
Q2 (11.70~17.31)	0.787 (0.635,0.977)	0.03	0.843 (0.678,1.046)	0.121	0.833 (0.667,1.041)	0.108
Q3 (17.31~24.98)	0.632 (0.503,0.793)	<0.001	0.699 (0.556,0.88)	0.002	0.714 (0.565,0.904)	0.005
Q4 (24.98~)	0.602 (0.478,0.758)	<0.001	0.760 (0.6,0.962)	0.023	0.775 (0.606,0.991)	0.042
p for trend		<0.001		0.005		0.014

Model a: No adjusted.

Model b: Adjusted for gender, age, and BMI.

Model c: Adjusted for gender, age, BMI, hypertension, diabetes, T stage, N stage, M stage, tumor size, perineural invasion, vascular invasion, differentiation, radiotherapy, chemotherapy.

### 3.6 Relationships between ANLR and sarcopenia/complication

For sarcopenia, multivariate analysis showed that each 1 SD increase in ANLR was associated with an 18.0% reduction in risk (OR = 0.820, 95% CI: 0.710–0.960, p = 0.009). The high-ANLR group had a 40.7% lower risk of sarcopenia than the low-ANLR group (Odds Ratio [OR] = 0.593, 95% CI: 0.442–0.796, p < 0.001). As ANLR increased, the OR for sarcopenia gradually decreased: compared with Q1, Q2 (0.816), Q3 (0.472), and Q4 (0.541) were all associated with a reduced risk of sarcopenia (**Table 3**).​ Regarding complications, each 1 SD increase in ANLR was linked to a 22.0% reduction in risk (OR = 0.780, 95% CI: 0.670–0.890, p < 0.001). The high-ANLR group had a 43.6% lower risk of complications than the low-ANLR group (OR = 0.564, 95% CI: 0.429–0.742, p < 0.001). With increasing ANLR, the HR for complications gradually decreased, with Q2 (0.561), Q3 (0.378), and Q4 (0.476) all associated with a reduced risk of adverse complications compared with Q1 (**Table 4**).

**Table 3:** Association between ANLR and sarcopenia of CRC patients.

ANLR	Model a	p value	Model b	p value	Model c	p value
Continuous (per SD)	0.789 (0.688,0.906)	<0.001	0.806 (0.699,0.93)	0.003	0.82 (0.71,0.96)	0.009
Cutoff value (High)	0.565 (0.433,0.737)	<0.001	0.592 (0.448,0.783)	<0.001	0.593 (0.442,0.796)	<0.001
Quartiles						
Q1 (~11.70)	ref		ref		ref	
Q2 (11.70~17.31)	0.816 (0.577,1.155)	0.251	0.789 (0.551,1.130)	0.196	0.849 (0.586,1.230)	0.387
Q3 (17.31~24.98)	0.472 (0.322,0.694)	<0.001	0.480 (0.323,0.714)	<0.001	0.494 (0.327,0.745)	<0.001
Q4 (24.98~)	0.541 (0.372,0.786)	0.001	0.568 (0.383,0.842)	0.005	0.597 (0.395,0.904)	0.015
p for trend		<0.001		<0.001		<0.001

Model a: No adjusted.

Model b: Adjusted for gender, age, and BMI.

Model c: Adjusted for gender, age, BMI, hypertension, diabetes, T stage, N stage, M stage, tumor size, perineural invasion, vascular invasion, differentiation, radiotherapy, chemotherapy.

**Table 4:** Association between ANLR and Complication of CRC patients.

ANLR	Model a	p value	Model b	p value	Model c	p value
Continuous (per SD)	0.756 (0.66,0.865)	<0.001	0.77 (0.67,0.884)	<0.001	0.780 (0.670,0.890)	<0.001
Cutoff value (High)	0.532 (0.412,0.689)	<0.001	0.555 (0.426,0.724)	<0.001	0.564 (0.429,0.742)	<0.001
Quartiles						
Q1 (~11.70)	ref		ref		ref	
Q2 (11.70~17.31)	0.561 (0.398,0.789)	<0.001	0.548 (0.387,0.774)	<0.001	0.542 (0.381,0.771)	<0.001
Q3 (17.31~24.98)	0.378 (0.262,0.548)	<0.001	0.388 (0.267,0.565)	<0.001	0.391 (0.267,0.574)	<0.001
Q4 (24.98~)	0.476 (0.334,0.676)	<0.001	0.494 (0.343,0.711)	<0.001	0.500 (0.343,0.730)	<0.001
p for trend		<0.001		<0.001		<0.001

Model a: No adjusted.

Model b: Adjusted for gender, age, and BMI.

Model c: Adjusted for gender, age, BMI, hypertension, diabetes, T stage, N stage, M stage, tumor size, perineural invasion, vascular invasion, differentiation, radiotherapy, chemotherapy.

### 3.7 Establishment of ANLR-based prediction nomograms

**Figure 4:**
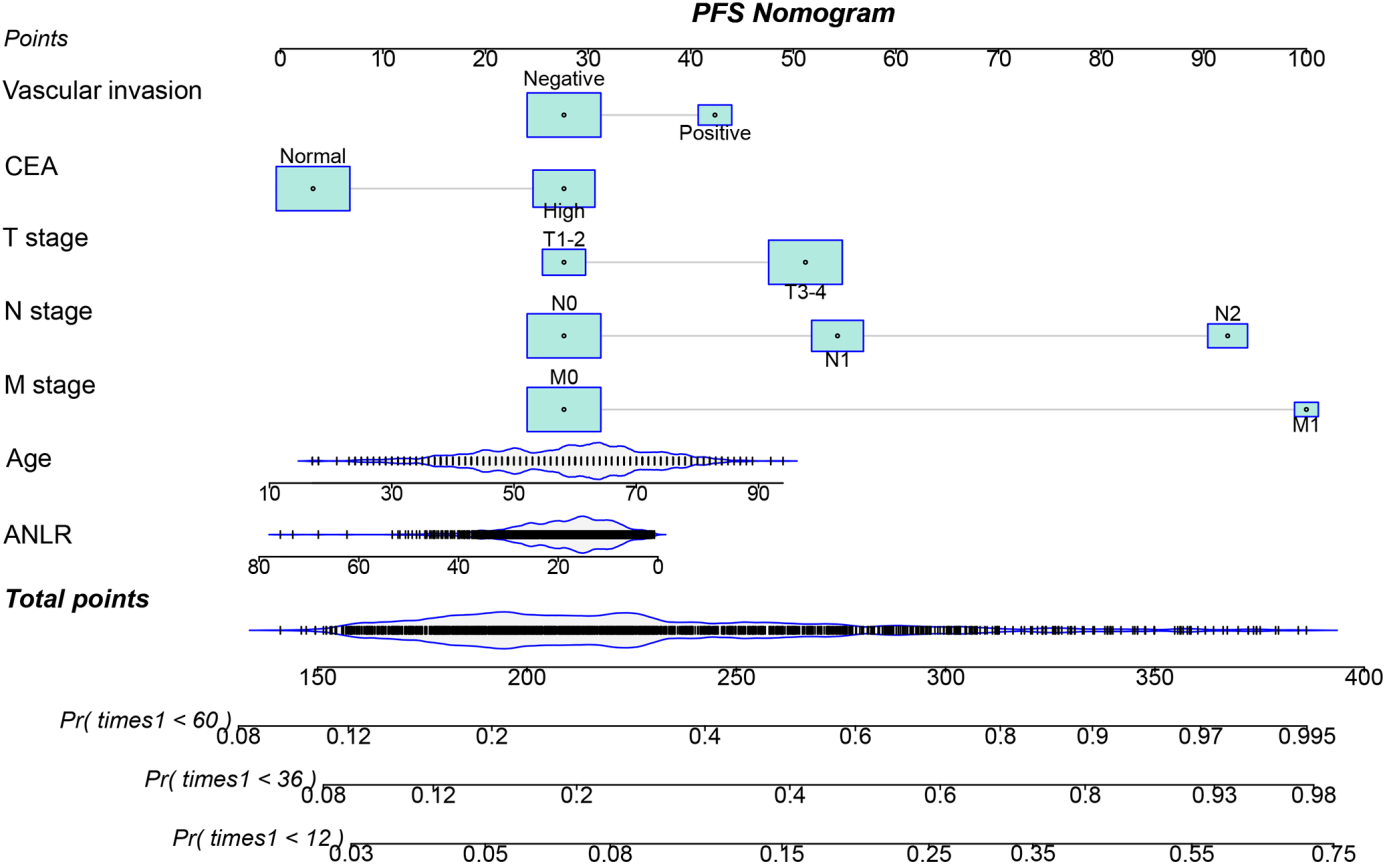
Construction of the PFS nomogram in CRC patients.

**Figure 5:**
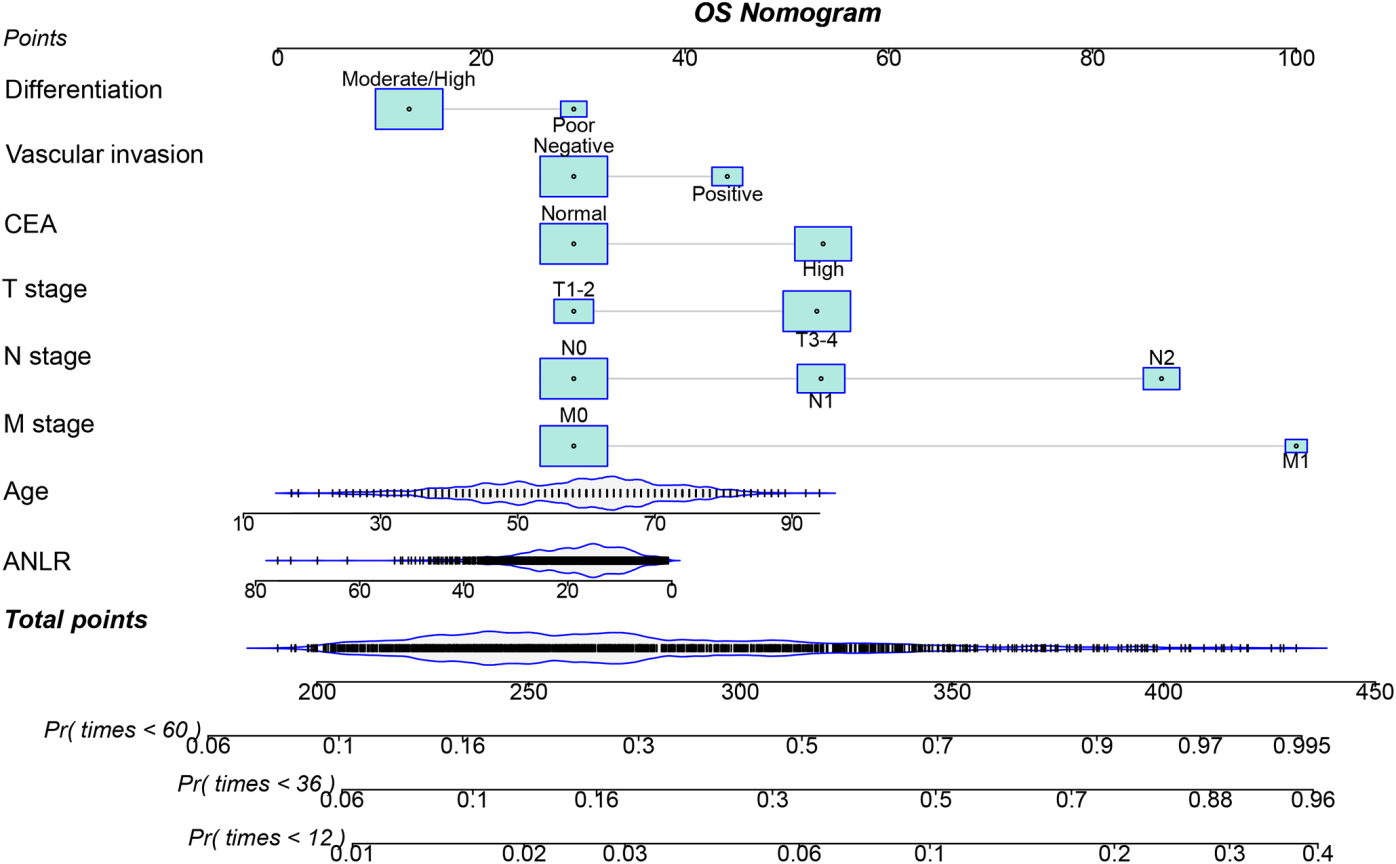
Construction the OS nomogram in CRC patients.

Multivariate Cox regression analysis identified seven independent prognostic factors influencing PFS in CRC patients: age, T stage, N stage, M stage, vascular invasion, CEA levels, and ANLR (**Supplementary Table 6**). For OS, the independent risk factors were age, T stage, N stage, M stage, vascular invasion, differentiation, CEA levels, and ANLR (**Supplementary Table 7**). The full coefficients of the Cox proportional hazards models for PFS and OS in the **Supplementary Tables 8**, **S9**. Based on these key factors, nomograms were developed to predict 1-, 3-, and 5-year PFS (**Figure 4**) and OS (**Figure 5**) in CRC patients. The predicted probabilities of PFS and OS at these time points were calculated by summing the scores assigned to each variable. To facilitate personalized prognostication for CRC patients, we deployed the PFS and OS nomogram models on a Shiny server, developing dedicated webpages for predicting PFS and OS in CRC patients. These webpages enable more convenient and individualized prognostic prediction for patients, with the URLs available at https://hailun.shinyapps.io/ANLR_PFS/(Accessed: [12/28/2025]) and https://hailun.shinyapps.io/ANLR_OS/(Accessed: [12/28/2025]) respectively (**Supplementary Table 10**).

The C-indices for the PFS and OS nomograms were 0.719 (95% CI: 0.698–0.740) and 0.727 (95% CI: 0.705–0.749), respectively. Calibration curves for 1–5-year postoperative PFS (**Supplementary Figure 7A**) and OS (**Supplementary Figure 7B**) demonstrated optimal consistency between the predicted survival probabilities and actual observed values. The 1-, 3-, and 5-year area under the ROC curve (AUC) values for the PFS nomogram were 0.802, 0.773, and 0.762, respectively, while those for the OS nomogram were 0.771, 0.774, and 0.762 (**Supplementary Figures 8A, B**). Patients were stratified into high- and low-score groups based on the median scores derived from the nomogram. Analysis showed that the high-score group had significantly poorer PFS and OS compared to the low-score group (**Supplementary Figure 9**). The DCA revealed that the ANLR-based nomograms provided greater clinical utility than the traditional TNM staging system for both PFS and OS at the 1-, 3-, and 5-year time points (**Supplementary Figures 10A, B**).

## 4 Discussion

This retrospective cohort study establishes the ANLR as a robust, independent prognostic biomarker in CRC, demonstrating significant clinical utility across multiple dimensions of patient outcomes. Our findings reveal that ANLR effectively stratifies CRC patients into distinct prognostic groups, with low ANLR strongly associated with adverse clinicopathological features including male sex, advanced age, larger tumor diameter, elevated CEA levels, and significantly worse survival outcomes. The striking survival discrepancy between the low and high ANLR groups in both PFS and OS underscores ANLR’s robust discriminative ability. Importantly, this prognostic superiority extends beyond traditional biomarkers, as evidenced by ANLR’s consistently higher AUC values compared to NLR, PLR, and PNI across multiple timepoints for both PFS and OS. This enhanced predictive performance likely stems from ANLR’s unique integration of systemic inflammation and nutritional status—two interconnected biological processes that collectively drive cancer progression through distinct yet complementary pathways.

The biological plausibility of ANLR as a superior biomarker lies in its dual reflection of critical cancer progression mechanisms. Neutrophils actively promote tumor angiogenesis and metastatic spread through matrix metalloproteinase secretion and immunosuppressive effects, while lymphopenia reflects compromised anti-tumor immunity (9, 13, 23–25). Simultaneously, albumin serves as both a nutritional reservoir and a negative acute-phase reactant; hypoalbuminemia indicates not only malnutrition but also hepatic reprioritization toward inflammatory protein synthesis during systemic inflammation (15, 16, 26). By synergistically capturing these pathways, ANLR provides a more comprehensive biological snapshot than single-dimension markers. This integrative capacity is further substantiated by our quartile analyses, which revealed a striking dose-dependent relationship between increasing ANLR levels and decreasing mortality risk—a pattern consistent across survival outcomes, sarcopenia development, and postoperative complications. Particularly compelling was the 26-44% risk reduction observed in high-ANLR patients across these clinical domains, suggesting ANLR’s value extends beyond survival prediction to encompass functional and treatment-related outcomes.

Clinically, ANLR’s consistent prognostic performance across all TNM stages and tumor locations enhances its practical utility. In early-stage (I-II) disease, the 9.9% absolute difference in 5-year OS between low and high ANLR groups could help identify candidates for treatment intensification, while in advanced (III-IV) CRC, the dramatic 17.4% OS gap might guide palliative strategy selection. Similarly, the 12.4% survival disadvantage for rectal cancer patients with low ANLR highlights its relevance in site-specific contexts where treatment approaches differ. Beyond survival metrics, ANLR’s strong inverse association with sarcopenia risk (18% reduction per SD increase) likely reflects albumin’s role in muscle protein synthesis and inflammation-driven catabolism, while its protective effect against complications (43.6% lower risk in high-ANLR group) aligns with evidence linking hypoalbuminemia to impaired wound healing and neutrophilia to tissue damage. These multidimensional correlations position ANLR as a holistic biomarker capable of informing both oncological and supportive care decisions.

The clinical translation of these findings is embodied in our ANLR-incorporated nomograms, which demonstrated excellent predictive accuracy and outperformed traditional TNM staging in decision curve analysis. The nomograms’ temporal validity and calibration precision support their implementation for personalized surveillance protocols, treatment modulation strategies, and resource allocation optimization. High-risk patients identified through these tools might benefit from intensified follow-up, nutritional interventions to address hypoalbuminemia, or even targeted anti-inflammatory therapies—though such applications require prospective validation.

Several study limitations warrant acknowledgment. The retrospective design of this study inherently carries the risk of selection bias and unmeasured confounding factors. For instance, data on adjuvant therapy heterogeneity (e.g., specific regimens, dose intensity) were not collected, which may impact PFS and OS and thus limit the comprehensiveness of our prognostic analysis. Additionally, potential confounders such as C-reactive protein, liver function indices, steroid use, and prehabilitation/nutrition interventions were not recorded, and their potential influence on ANLR and patient outcomes cannot be ruled out. Furthermore, the ANLR-based prediction nomograms are internally derived, constructed from a single-center cohort. Despite their demonstrated predictive performance, external validation in prospective, multi-center studies is required to confirm their clinical applicability. The precise biological mechanisms linking ANLR to sarcopenia deserve further exploration through serial measurements during treatment. Future research should prioritize validating ANLR in non-Asian populations, investigating its dynamic changes during therapy as a potential response marker, and developing ANLR-guided intervention trials to determine whether modulating this composite index improves clinical outcomes.

## 5 Conclusion

This comprehensive analysis establishes ANLR as a clinically potent, readily calculable biomarker that integrates the critical prognostic dimensions of inflammation and nutrition in colorectal cancer. ANLR demonstrates superior prognostic accuracy compared to established indices for predicting survival outcomes while extending its predictive value to functional sequelae like sarcopenia and treatment complications. The consistent performance across disease stages and tumor locations underscores its broad applicability in routine practice. Our developed ANLR-based nomograms provide clinicians with practical tools for individualized risk assessment, potentially enhancing treatment decision-making and resource allocation. Implementation of ANLR in clinical pathways could optimize risk stratification, guide therapeutic intensification, and ultimately improve both oncological and functional outcomes for CRC patients.

## Data Availability

The original contributions presented in the study are included in the article/**Supplementary Material**, further inquiries can be directed to the corresponding authors.
